# Mediating Factors in Within-Person Developmental Cascades of Externalising, Internalising and ADHD Symptoms in Childhood

**DOI:** 10.1007/s10802-022-00905-5

**Published:** 2022-04-30

**Authors:** Lydia Gabriela Speyer, Ingrid Obsuth, Denis Ribeaud, Manuel Eisner, Michelle Luciano, Bonnie Auyeung, Aja Louise Murray

**Affiliations:** 1 Department of Psychology, University of Edinburgh, Edinburgh, UK; 2 Department of Psychology, University of Cambridge, Cambridge, UK; 3 Clinical Psychology Department, University of Edinburgh, Edinburgh, UK; 4 Violence Research Centre, Institute of Criminology, University of Cambridge, Cambridge, UK; 5 Jacobs Center for Productive Youth Development, University of Zurich, Zurich, Switzerland; 6 Autism Research Centre, Department of Psychiatry, University of Cambridge, Cambridge, UK

**Keywords:** Developmental cascades, Internalising, Externalising, ADHD Symptoms, z-proso

## Abstract

Previous studies have offered evidence for peer problems and academic achievement as mediators in developmental cascades from externalising to internalising problems, and from ADHD symptoms to both internalising and externalising problems. However, these mediators have not been found to fully account for these cascades, indicating that there may be additional mediators involved. This study investigated the role of harsh parenting and parental involvement alongside academic achievement and peer problems in mediating within-person developmental cascades from externalising to internalising problems and from ADHD symptoms to internalising and externalising problems using autoregressive latent trajectory models with structured residuals. Models were fit for parent- and teacher-reports on children’s psychosocial development as measured by the Social Behaviour Questionnaire (SBQ) collected over ages 7, 9, and 11 in an ethnically diverse Swiss longitudinal cohort study (z-proso; *N* = 1387, 51% male). Results indicated that, when appropriately disentangling within- from between-person effects, none of the considered factors acted as significant mediators in longitudinal within-person relations between ADHD, internalising and externalising problems; hence, mediating mechanisms in developmental cascades remain to be identified.

**Supplementary Information:**

The online version contains supplementary material available at 10.1007/s10802-022-00905-5.

According to the World Health Organisation (WHO, 2018) up to 20% of children and adolescents globally suffer from a mental health problem and about 50% of all mental health conditions have their onset by age 14 (Kessler et al., 2005). The leading mental health concerns among young people are Attention Deficit Hyperactivity Disorder (ADHD), behaviour (or externalising) problems, and anxiety and depression (collectively ‘internalising problems’) (CDC, 2021; Danielson et al., 2018; Perou et al., 2013). These issues commonly co-occur (Cunningham & Ollendick, 2010; Gnanavel et al., 2019; Reale et al., 2017), making it important to understand the processes through which different childhood mental health problems are linked over time. To add to this understanding, in this study we examined the roles of academic achievement, peer problems and two parenting factors: harsh parenting and parental involvement, as potential mechanisms linking ADHD symptoms, internalising problems, and externalising problems. Importantly, we model the within-person processes that are likely of primary interest to interventions aiming to reduce the co-occurrence of mental health problems (Hamaker et al., 2015).

Developmental cascade models hypothesise that links between mental health domains are the result of problems in one domain causally affecting the other domain, potentially through a number of linking mechanisms (Masten & Cicchetti, 2010). According to the dual failure model, for example, externalising problems lead to the development of internalising problems through unidirectional cascades from the former to the latter via educational underachievement and social difficulties such as peer problems (Capaldi, 1992). While a number of studies have found evidence for cascade effects from externalising to later internalising problems, e.g., from middle childhood to early adolescence (Moilanen et al., 2010) or during adolescence (e.g., Blain-Arcaro & Vaillancourt, 2017) as well as for a mediating effect of peer relationship and academic achievement (e.g., van Lier et al., 2012), these linking factors have not been found to fully explain externalising-internalising cascades (e.g. Evans & Fite, 2019; Poirier et al., 2019). Given the complexity of the development of mental health problems, it is highly likely that other factors also play a mediating role in these cascades. In particular, to date, few studies have investigated the role of parenting behaviours as a potential mechanism for linking externalising-internalising cascades, even though Capaldi’s (1992) originally hypothesised dual failure model explicitly included parental relationships. Also, other developmental psychopathology models and in particular Patterson’s coercive parenting model (1982) have explicitly highlighted the role of parenting behaviours in escalating externalising problems through a coercive cycle of parent–child interaction. Evidence for such coercive family processes has been mixed (Besemer et al., 2016; Lansford et al., 2012), however, there is substantial evidence highlighting that maladaptive parenting practices are associated with both internalising and externalising problems (for reviews see Pinquart, 2017a, b). Consequently, parenting behaviours are a strong candidate for linking the developmental relations of externalising and internalising problems. One study that included a parental factor in a developmental cascade model focusing on middle to late childhood found that maternal dissatisfaction with their child was a significant mediator in a developmental cascade from externalising problems at age 5 to internalising problems at age 12 (Wertz et al., 2015).

Two parental factors that may be particularly relevant in mediating developmental cascades are harsh parenting and parental involvement. Many studies have suggested that harsh parenting practices lead to increased behavioural problems (for a review, see Gershoff, 2002; Pinquart, 2017a) with research further pointing to internalising problems as an outcome of the coercive parent–child interaction cycle proposed by Patterson (Dallaire et al., 2010; Pinquart, 2017b; Speyer et al., 2022). Other research has further suggested that parental involvement may be relevant in the development of both externalising and internalising problems (Aboobaker et al., 2019; Hawes et al., 2013; Kirkhaug et al., 2013). For instance, using the Alabama Parenting Questionnaire which measures six domains of parenting behaviour, Aboobaker et al. (2019) found that, out of all measured parenting domains, parental involvement was the only parenting domain associated with emotional problems in a sample of 10 to 18 year olds. Considering the limited research on the role of parenting factors in developmental cascades from externalising to internalising problems, further research is needed in order to gain a more comprehensive understanding of the mechanisms that may lead to the high co-occurrence of mental health problems.

Internalising and externalising problems also commonly co-occur with ADHD, with up to 50% of children experiencing ADHD symptoms also experiencing co-occurring internalising or externalising problems (Gnanavel et al., 2019; Reale et al., 2017). While ADHD was previously classified as a disruptive behaviour disorder following DSM-IV-TR diagnostic criteria, and as a behavioural and emotional disorder following ICD-10 criteria, it is now considered to be a neurodevelopmental disorder in the most recent diagnostic guidelines (i.e. DSM-5 and ICD-11). Thus, while discussions on whether ADHD should be viewed as an externalising problem or as a distinct entity are ongoing, for the purposes of the current study, we follow current diagnostic guidelines that place ADHD outside externalising problems. This allows for the possibility that cascades involving ADHD symptoms differ from those involving issues such as aggressive, oppositional, and conduct problems.

One candidate explanation for the high co-occurrence of ADHD and externalising problems is offered by Beauchaine and McNulty’s (2013) ontogenic process model of externalising psychopathology which hypothesises that externalising problems are a result of longitudinal transactions between contextual risk factors and individual vulnerabilities (e.g. genetic factors). The theory implies that heritable traits such as impulsivity may lead some individuals to traverse the externalising spectrum as such traits set them up for contextual problems such as coercive parent–child interactions, poorer peer relationships and academic underachievement, leading to a developmental trajectory characterised by escalating externalising behavioural problems. Thus, the ontogenic process model of externalising psychopathology would suggest that ADHD symptoms, impulsivity in particular, leads to increased externalising problems over time through similar mechanism as hypothesised by Patterson’s coercion model or the dual failure model (Ahmad & Hinshaw, 2016). A large number of longitudinal studies have found evidence for such processes, finding that childhood ADHD symptoms were associated with difficulties in the family environment such as parenting stress (Theule et al., 2013), and preceded conduct problems (Gustafsson et al., 2018), antisocial personality disorder (Klein et al., 2012), peer problems (Diamantopoulou et al., 2005), educational underachievement (Barry et al., 2002), delinquency (Mannuzza et al., 2008) as well as substance use (Molina et al., 2018). With regards to mediating and moderating factors, prior longitudinal research has suggested that school performance may mediate the associations between childhood ADHD and adverse life outcomes such as adolescent alcohol use, while parenting factors, such as parental knowledge, may play a moderating role in that association (Molina et al., 2012). However, few studies to date have investigated the processes implied by the ontogenic process models within a developmental cascade framework. Doing so would allow for better insights into the mechanisms that link ADHD symptoms and externalising problems over time, especially when investigated within a comprehensive framework that allows for the investigation of multiple inter-related factors within one model. One of the few studies to date that has investigated developmental cascades within such an integrated framework found that ADHD symptoms were associated with increased peer problems, conduct problems as well emotional problems across ages 3 to 17 (Speyer et al., 2021b), thus, suggesting that ADHD symptoms play a particularly important role for the development of later psychosocial difficulties. With regards to investigations into mediating mechanisms such as the mechanism implied by Patterson’s coercion model or the dual failure model, very little research has investigated such processes within a developmental cascade framework (e.g. Sevincok et al., 2020). Further research from a developmental cascades perspective is thus needed to help illuminate how problems in one domain might engender problems in others over time.

Longitudinal investigations of the relations between ADHD and internalising problems have also found support for developmental cascades from ADHD symptoms to internalising problems as well as for cascades in the opposite direction, both in middle to late childhood (Speyer et al., 2021a) as well as in middle adolescence (Murray et al., 2020a) and across early childhood to late adolescence (Speyer et al., 2021b). A number of studies have applied variants of the dual failure model to ADHD-internalising cascades, finding support for mediating effects of peer problems and academic attainment (Powell et al., 2020; Roy et al., 2015). Some evidence also suggests that parental relationships or ineffective behavioural management may act as links between ADHD symptoms and depression (Humphreys et al., 2013; Ostrander & Herman, 2006).

One limitation common to most studies on developmental cascades; however, relates to their statistical operationalisation. The most frequently used method to investigate developmental cascades and potential mediating factors is the cross-lagged panel model (CLPM), which examines the relations between variables over time after adjusting for inter-individual stability in those same variables. However, the CLPM does not separate between-person differences from within-person relations, which means that its parameters represent a mixture of within-and between-person effects that provide ambiguous results (Berry & Willoughby, 2017). Considering that developmental cascades refer to within-person processes, more appropriate methods are needed to confirm that the established relations between externalising problems as well as ADHD symptoms and internalising problems still hold when partialling out between-person differences. One modelling technique well-suited to overcome this limitation of the CLPM, is the autoregressive latent trajectory model with structured residuals (ALT-SR) (Curran et al., 2014). ALT-SRs combine the CLPM with a parallel process latent growth curve model that accounts for between-person effects through allowing growth factor intercepts of different variables to covary. Relatively few studies have applied ALT-SRs to the study of developmental cascades (Murray et al., 2020b; Oh et al., 2020), and even fewer to the study of potential mediators (Murray et al., 2021). The only study on mediating factors using an ALT-SR so far has not provided strong evidence for the dual failure model. Using data from the same cohort as the current study, Murray et. al (2021) investigated the mediating role of peer- and teacher-relationships in developmental cascades between self-reported aggression and internalising problems during adolescence. Murray et al. did not identify any significant mediation effects nor did they find support for developmental cascades between aggression and internalizing problems. However, they only investigated these relations during adolescence (ages 11, 13 and 15) and did not consider the role of parental involvement or academic achievement as well as ADHD symptoms in these relations. Since previous research has shown that the within-person developmental relations between internalising and externalising problems unfold differently in childhood compared to adolescence (Murray et al., 2020b), it is important to also investigate mediators in the dual-failure model during childhood.

In the current study, we aimed to comprehensively investigate the within-person relations between some of the most commonly co-occurring mental health problems in childhood. In particular, we aimed to extend the dual failure model by investigating whether within-person developmental cascades from externalising problems to internalising problems as well as cascades from ADHD symptoms to internalising and externalising problems during childhood (median-ages 7, 9 and 11) were mediated by harsh parenting, parental involvement, peer problems and academic achievement. Peer problems were operationalised as negative social roles in the classroom, such as being a bully or a bullying victim. These have previously been identified to be associated with internalising as well as externalising problems (Kelly et al., 2015). In addition, children with multiple negative social roles (e.g., being a bully and a bullying victim) have been found to have more internalising and externalising problems than children with just one negative role (being a bully or a bullying victim) (Kelly et al., 2015). Based on the considerations discussed above, we used an autoregressive latent trajectory (ALT-SR) model with structured residuals to achieve insights into the within-person dynamics of the studied developmental relations. Since previous research has highlighted the need for multi-informant approaches to the studies of children’s psychosocial development (De Los Reyes, 2013) we fit ALT-SRs using parent-reported data on internalising, externalising and ADHD symptoms as well as using teacher-reported data to evaluate the stability of our results across informants. This was deemed to be particularly important since previous research using the ADHD data used in the current study already highlighted that agreement on children’s ADHD symptom trajectories was only low to moderate across parents and teachers (Murray et al., 2018, 2019c).

## Methods

### Participants

Participants were 1,387 children who were part of the Zurich Project on Social Development from Childhood to Adulthood (z-proso), a Swiss longitudinal cohort study. Z-proso has been following the lives of approximately 1,500 children of a target sample of 1,675 children from 2004 when the children entered school at age 7 up until the present day, with data collection still ongoing. Participants attended one of 56 schools in Zurich that were chosen based on a stratified random sampling procedure that included the school’s size and location as stratification variables to ensure representativeness in terms of area-based deprivation. While the official language of the canton of Zurich is German, Zurich is a very culturally diverse city, thus, in order to recruit and retain as many of the non-German speaking participants as possible, contact letters and parent interviews were translated into an additional nine languages. In the current sample, 51% of participants were male with sex established based on the sex assigned at birth as recorded in the database of school authorities. School authorities received this information from the local population authority which in turn gets information on any new-born child’s sex directly from the clinic in which the child is born. For children moving from one area within Switzerland to another, information on the child’s sex assigned at birth is shared between the different local population authorities, while for children immigrating from another country, sex is established based on passport information. For an overview of key sample demographics, see Table 1 and for a more detailed breakdown of demographics, see Table S1 in the online supplementary. Information on clinical diagnoses was not collected in z-proso, however, around 5% of participants self-reported using medications that are typically prescribed for the treatment of ADHD symptoms (Murray et al., 2018). Further details on assessment procedures, recruitment, and attrition can be found in the literature (Ribeaud et al., 2022) and on the study website: https://www.jacobscenter.uzh.ch/en/research/zproso/aboutus.html. Ethical approval for z-proso was obtained from the University of Zurich’s Ethics Committee from the Faculty of Arts and Social Sciences. Active informed consent for children to participate was provided by parents. This study uses data from waves at median-ages 7, 9 and 11 since at these ages comparable data on internalising, externalising and ADHD symptoms as well as on peer problems, academic achievement and parental involvement were available.

**Table 1:** Sample demographic information at baseline (age 7)

**Variable**	**Category**	**%**	**N**
**Child Sex**	Female	48.1	596
	Male	51.9	644
**Language Spoken at Home: Child**	Swiss German	50.2	624
	Other	49.8	616
**Country of Birth: Child**	Switzerland	89.4	1109
	Other	10.6	131
**Academic Qualification:**	Mandatory school or less	21.0	213
**Male Primary Caregiver**	Apprenticeship	27.2	276
	A-levels	9.7	99
	Higher vocational education	17.1	174
	University	25.0	254
**Academic Qualification:**	Mandatory school or less	25.4	309
**Female Primary Caregiver**	Apprenticeship	35.2	428
	A-levels	7.8	95
	Higher vocational education	15.5	189
	University	16.0	195

### Measures

#### Psychosocial Development

Internalising problems, externalising problems and ADHD symptoms were measured using the Social Behaviour Questionnaire (SBQ; Tremblay et al., 1991) which assesses children’s psychosocial development across five domains: aggression, non-aggressive externalizing problems, anxiety/depression, ADHD symptoms and prosocial behaviour. At median-ages 7, 9 and 11, teachers completed a German paper-and-pencil version of the SBQ while parents completed a computer-assisted personal interview (Murray et al., 2019b). Most of the interviews were conducted in German, but parents were able to complete the interview in an additional nine languages. Items in the paper-and-pencil version of the SBQ were presented in the same order to all teachers, whereas the computer assisted parent interview presented items in a randomised order. Self-reported SBQs were also available, but for ages 7, and 9, these were collected in the form of an adapted computer-based multimedia version of the SBQ with children answering ‘yes’ or ‘no’ to a series of questions relating to their psychosocial development while from age 11 onwards, they were collected using a paper-and-pencil version of the SBQ with answers given on a Likert scale in line with parent- and teacher-reported SBQs. Given the lack of continuity of measurement, we did not include self-reports in the current study. Internalising problems (e.g., < child name > appears miserable, distressed, or unhappy) were measured using four items on depression and three items on anxiety. All items were rated on a 5-point Likert scale from *Never* to *Very Often* and subsequently averaged to create a composite score (parent-report: Cronbach’s α_age 7_ = 0.71; α_age 9_ = 0.76; α_age 11_ = 0.79; teacher-report: Cronbach’s α_age 7_ = 0.89; α_age 9_ = 0.91; α_age 11_ = 0.91). Higher scores indicate more mental health problems. Composite scores for externalising problems (e.g., < child name > reacts in an aggressive manner when teased) were created by averaging item scores of four items related to non-aggressive conduct problems, two items measuring symptoms of oppositional defiant disorder, five items on physical aggression, three items on proactive aggression and 3 items on reactive aggression, thus, reflecting the multi-dimensional nature of externalising problems (parent-report: Cronbach’s α_age 7_ = 0.81; α_age 9_ = 0.82; α_age 11_ = 0.84; teacher-report: Cronbach’s α_age 7_ = 0.94; α_age 9_ = 0.94; α_age 11_ = 0.94). The ADHD symptom (e.g., < child name > is impulsive, acts without thinking) composite score was derived from four items on inattention and four items on hyperactivity/impulsivity (parent-report: Cronbach’s α_age 7_ = 0.79; α_age 9_ = 0.84; α_age 11_ = 0.85; teacher-report: Cronbach’s α_age 7_ = 0.94; α_age 9_ = 0.95; α_age 11_ = 0.95). Psychometric analyses of the SBQ in the current sample have found support for developmental invariance, factorial and criterion validity of scores and have shown that the SBQ provides scores that are a reliable measure of moderately low to very high levels of psychopathology in the general population (Murray et al., 2017, 2019b). A factor analytic study of the SBQ has further found support for keeping ADHD items separate from externalising problems as they did not fit well within the externalising domain (Murray et al., 2019b).

#### Mediating Factors

Academic achievement was measured using a composite score of arithmetic skills and reading and verbal language performance scores. Considering that teachers are best placed to rate children’s academic performance, teachers were asked to compare children’s abilities in arithmetic and language performance to an average student of the same age on a five-point Likert scale from ‘worse’ to ‘better than average’. These scores were averaged to create a composite score with higher scores indicating better academic performance (Cronbach’s α_age 7_ = 0.79; α_age 9_ = 0.80; α_age 11_ = 0.83).

Peer problems were also teacher-reported and were based on a measure of children’s social roles in the classroom. Negative roles in the classroom have been identified to be related to both internalising and externalising problems and can naturally be best observed by teachers. Teachers were asked to rate the child’s role on four items relating to popularity, bullying victimisation, rejection and domination on a 5-point Likert scale from ‘very untrue’ to ‘very true’ (e.g., < child name > dominates others). Before averaging all items to create a composite score, the item on popularity was reverse coded in order to be consistent with the other items. Higher scores indicate that a child has more negative social roles in the classroom, thus more peer problems (Cronbach’s α_age 7_ = 0.67; α_age 9_ = 0.73; α_age 11_ = 0.69).

Parental involvement and harsh parenting were measured using the Alabama Parenting Questionnaire (APQ) which measures parenting behaviours in six domains: parental involvement, positive parenting, poor monitoring, inconsistent discipline, corporal punishment, and other discipline (Shelton et al., 1996). The involvement subscale includes ten items (e.g., You talk with < child name > about his/her friends) that parents rated on a 5-point Likert scale ranging from ‘never’ to ‘always’. Scores on all items were averaged to create a composite score (Cronbach’s α_age 7_ = 0.63; α_age 9_ = 0.67; α_age 11_ = 0.72) with higher scores being indicative of more parental involvement. Harsh parenting was measured using the corporal punishment subscale which consisted of ten items (e.g., You slap < child name > when he/she has done something wrong) that were averaged with higher scores indicating more incidents of harsh parenting (Cronbach’s α_age 7_ = 0.53; α_age 9_ = 0.55; α_age 11_ = 0.62).

### Statistical Analysis

Autoregressive Latent Trajectory Models with Structured Residuals (ALT-SR) were fitted to investigate the within-person effects of academic achievement, peer problems and parenting factors on developmental cascades from externalising problems and ADHD symptoms to internalising problems. In order to keep the complexity of the ALT-SRs to a minimum, a first model was built for parental involvement as a mediator alongside peer problems and academic achievement, and a second for investigating the effect of harsh parenting alongside peer problems and academic achievement. Before introducing mediators to our model, we also fit ALT-SRs for the psychopathology variables only in order to be able to examine whether adding mediators changed any associations observed in the baseline psychopathology model. Models were built separately for parent- and teacher-reported data on children’s psychosocial development since teacher- and parent-reports on children’s psychosocial functioning tend to show limited convergence with cross-informant reports correlating at a low-to-moderate magnitude (De Los Reyes et al., 2015). Additionally, combining an ALT-SR with a latent variable model for teacher and parent reported mental health data would have likely resulted in estimation difficulties. However, as a sensitivity analysis, we also built ALT-SRs with parent and teacher reports on psychosocial difficulties combined based on averaging symptom scores across informants.

ALT-SRs combine cross-lagged panel models with latent growth curve models to optimally account for between-person effects which in the context of developmental cascade models act more as a confound than as effects of substantiative interest. The latent growth curve part of the model included random intercepts that are allowed to co-vary and fixed slopes, thus capturing stable between-person differences (e.g., differences in parental education) in baseline levels of included variables. Slope factor loadings were fixed proportional to the time intervals between measurement occasions. The autoregressive and cross-lagged effects are defined between the variables’ residuals, reflecting deviations from the person-specific means and growth curves at certain time points and, thus, offering insights into within-person dynamics. The cross-lagged part of the model included all first-order autoregressive and cross-lagged effects as well as within-time residual covariances. In addition, mediation effects were estimated for cascades from externalising to internalising problems via peer problems, parental involvement/harsh parenting and academic achievement as well as for ADHD symptoms to internalising and externalising problems via the same potential mediators. Second-order cross-lagged effects between externalising at time 1 and internalising problems at time 3 as well as between ADHD at time 1 and internalising and externalising at time 3 were further included to allow for testing of longitudinal mediation. To assess statistical significance of estimated indirect effects, standard errors were computed using the delta method. However, since the delta method incorrectly assumes a symmetric sampling distribution of indirect effects (MacKinnon et al., 1995), we also computed bootstrapped 95% confidence intervals using standard maximum likelihood estimation.

Since previous research has indicated that mental health development differs by child sex (Lewis et al., 2020; Murray et al., 2019a), we also fitted multi-group ALT-SRs with autoregressive and cross-lagged paths constraint to be equal across sexes in order to examine whether sex moderated the observed associations. Model comparisons based on Baysian Information Criterion (BIC) suggested that, across all analyses, the constraint models did not fit better than the unconstraint models (i.e., with only the necessary constraints for identification imposed), thus we proceeded with a combined model for both sexes. Sex was further regressed onto each intercept factor to adjust for any potential sex differences in baseline levels of difficulties.

Models were fit in Mplus 8.5 (Muthén & Muthén, 2018) using a robust maximum likelihood estimator (MLR) which uses full information maximum likelihood (FIML) to handle missing data (Enders, 2001). Model fit was judged to be acceptable based on the following criteria: Tucker Lewis Index (TLI) > 0.90, Comparative Fit Index (CFI) > 0.90 and Root Mean Squared Error of Approximation (RMSEA) < 0.05 (Kline, 2005). Mplus code and full results for all conducted analyses including information on developmental trajectories are provided on the OSF: https://osf.io/d7wfb/.

## Results

### Descriptive Statistics

Descriptive statistics are provided in Table S2 in the online supplementary. Correlations between parent- and teacher-reported variables on psychosocial development were relatively small, ranging from 0.11 to 0.22 for internalising problems, from 0.19 to 0.23 for externalising problems and from 0.36 to 0.42 for ADHD symptoms. Correlations between the different SBQ subscales ranged from 0.18 (internalising age 7 – ADHD age 11) to 0.53 for parents (ADHD age 9 – externalising age 9) and from 0.06 (internalising age 7 – externalising age 11) to 0.69 (ADHD age 9 – externalising age 9) for teachers. For a correlation matrix of all observed variables, see the online supplementary Table S3.

### Parent-Reported Model Without Mediators

**Fig. 1:**
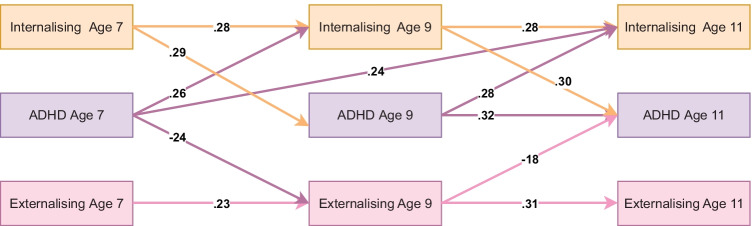
Standardised autoregressive and cross-lagged parameters from baseline ALT-SR without mediators using parent-reported data. Int = internalising, ADHD = ADHD symptoms, Ext = Externalising. Only statistically significant paths are shown. Latent growth curves (inlcuding random intercepts that are allowed to covary and fixed slopes) and residual covariance parameters are omited for clarity

The baseline model for parent-reported psychopathology variables fit well (RMSEA = 0.059, CFI = 0.991 and TLI = 0.953) and suggested that ADHD and internalising problems share reciprocal relations while ADHD symptoms at age 7 were associated with decreased externalising problems at age 9 which in turn were associated with decreased ADHD symptoms at age 11. Figure 1 shows a summary of significant standardised autoregressive and cross-lagged parameters. Full model results are available on OSF: https://osf.io/d7wfb/

### Teacher-Reported Model Without Mediators

**Fig. 2:**
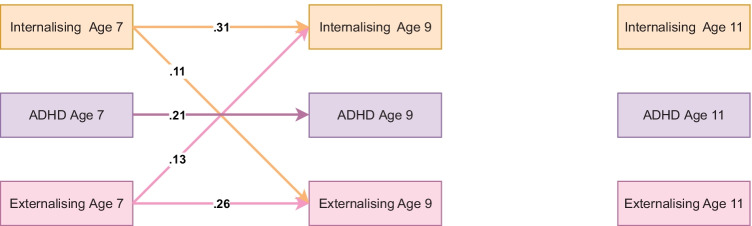
Standardised autoregressive and cross-lagged parameters from baseline ALT-SR without mediators using teacher-reported data. Int = internalising, ADHD = ADHD symptoms, Ext = Externalising. Only statistically significant paths are shown. Latent growth curves (inlcuding random intercepts that are allowed to covary and fixed slopes) and residual covariance parameters are omited for clarity

The teacher-reported baseline model fit equally well (RMSEA = 0.075, CFI = 0.981, TLI = 0.907), but, in contrast to the parent-reported baseline model suggested that internalising and externalising problems shared reciprocal relations between age 7 and age 9. A summary of significant standardised autoregressive and cross-lagged parameters is available in Fig. 2 and full results are available on OSF: https://osf.io/ms8q9/

### Parent-Reported Model Including Parental Involvement

**Fig. 3:**
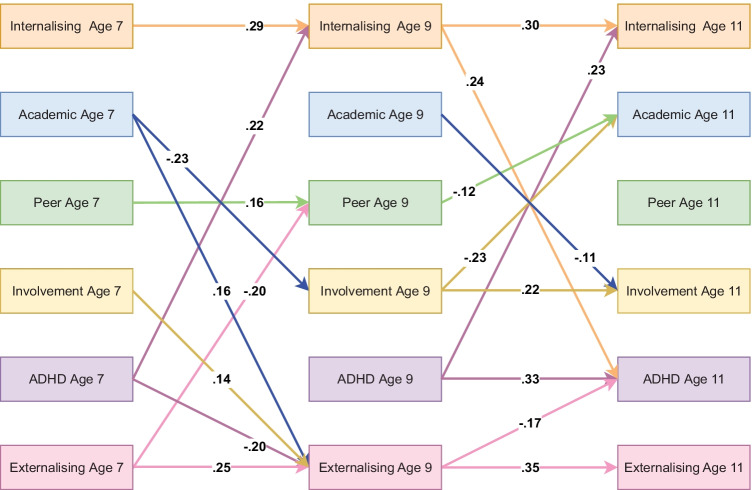
Standardised autoregressive and cross-lagged parameters from ALT-SR for parental involvement using parent-reported data. Int = internalising, Academic = academic achievement, Peer = peer problems, Involvement = parental involvement, ADHD = ADHD symptoms, Ext = Externalising. Only statistically significant paths are shown. Latent growth curves (inlcuding random intercepts that are allowed to covary and fixed slopes) and residual covariance parameters are omited for clarity

Model fit indices for the ALT-SR using parent reported data showed good fit with RMSEA = 0.038, CFI = 0.992 and TLI = 0.954. For a summary of significant standardised autoregressive and cross-lagged parameters see Fig. 3. In terms of cross-lagged effects, ADHD symptoms at age 7 and at age 9 were found to be predictive of internalising problems at age 9 and age 11 respectively. Age 9 internalising was also predictive of age 11 ADHD, thus showing evidence for a reciprocal relation between ADHD and internalising difficulties during late childhood. The model further identified a significant cascade from ADHD symptoms at age 7 to externalising problems at age 9 and from age 9 externalising to age 11 ADHD. Results did not indicate any statistically significant cascades from externalising to internalising problems. Analyses of indirect effects indicated that neither cascades from externalising to internalising problem nor cascades from ADHD symptoms to internalising or externalising problems were significantly mediated by peer problems, academic achievement or parental involvement, see Table S4(a) in the online supplementary. Their combined mediating effect was also not statistically significant which was further confirmed using bootstrapped confidence intervals. For full results, please refer to https://osf.io/z38x4/.

### Teacher-Reported Model Including Parental Involvement

**Fig. 4:**
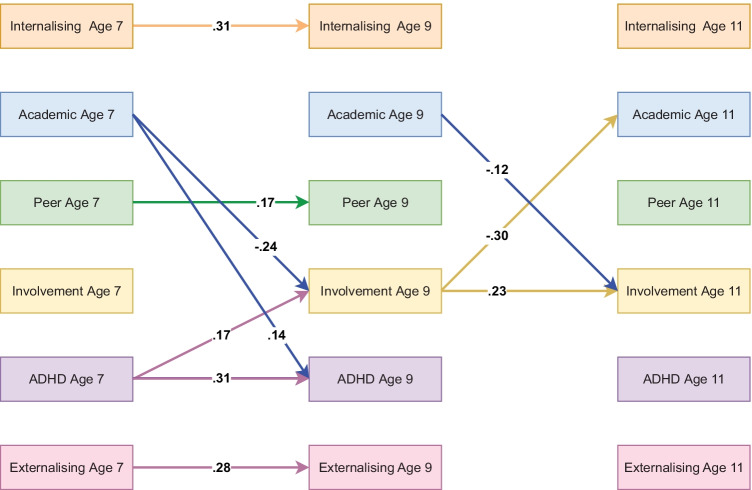
Standardised autoregressive and cross-lagged parameters from ALT-SR for parental involvement using teacher-reported data. Int = internalising, Academic = academic achievement, Peer = peer problems, Involvement = parental involvement, ADHD = ADHD symptoms, Ext = Externalising. Only statistically significant paths are shown. Latent growth curves (inlcuding random intercepts that are allowed to covary and fixed slopes) and residual covariance parameters are omited for clarity

The teacher-reported ALT-SR including parental involvement also showed good fit (RMSEA = 0.050, CFI = 0.987, TLI = 0.927). Significant standardised autoregressive and cross-lagged parameters are visualised in Fig. 4. In contrast to the parent-reported model, no significant developmental cascades involving psychosocial difficulties were identified but, in line with the parent-reported model, neither externalising-internalising cascades nor ADHD-internalising or ADHD-externalising cascades were significantly mediated by peer problems, academic achievement or parental involvement. Their combined mediating effect was also not statistically significant based on bootstrapped confidence intervals, see Table S4(b) in the online supplementary. For full results, please see here: https://osf.io/egrjc/.

### Parent-Reported Model Including Harsh Parenting

**Fig. 5:**
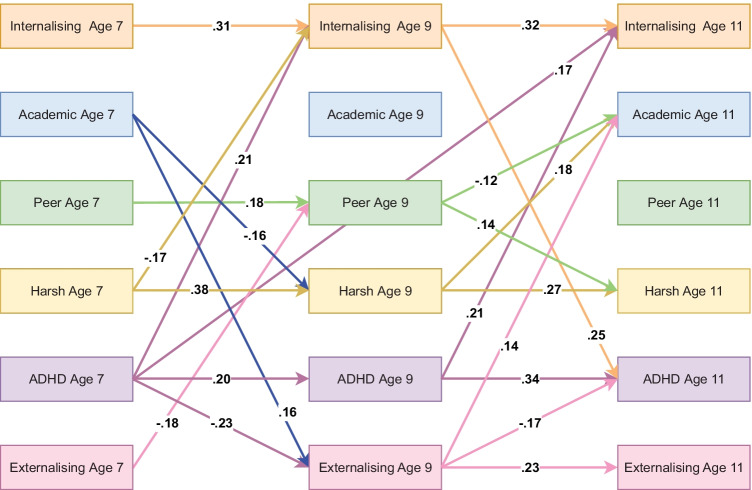
Standardised autoregressive and cross-lagged parameters from ALT-SR for harsh parenting using parent-reported data. Int = internalising, Academic = academic achievement, Peer = peer problems, Harsh = harsh parenting, ADHD = ADHD symptoms, Ext = Externalising. Only statistically significant paths are shown. Latent growth curves (inlcuding random intercepts that are allowed to covary and fixed slopes) and residual covariance parameters are omited for clarity

The model including harsh parenting using parent-reported data fit well (RMSEA = 0.045, CFI = 0.988, TLI = 0.931). This model yielded essentially the same results as the parent-reported model including parental involvement with no significant mediation effects being identified. However, this model suggested that ADHD symptoms at age 7 were not only predictive of internalising problems at age 9 but also at age 11. Harsh parenting at age 7 further was associated with decreased internalising problems at age 9 and, at age 9 with lower academic achievement at age 11. Significant autoregressive and cross-lagged effects are summarised in Fig. 5, indirect effects are available in Table S4(c) in the online supplementary and full results are available on the OSF: https://osf.io/cr48d/.

### Teacher-Reported Model Including Harsh Parenting

**Fig. 6:**
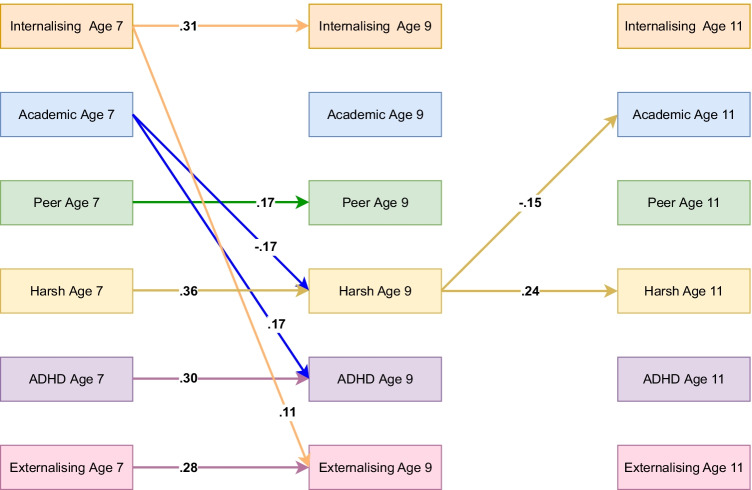
Standardised autoregressive and cross-lagged parameters from ALT-SR for harsh parenting using teacher-reported data. Int = internalising, Academic = academic achievement, Peer = peer problems, Harsh = harsh parenting, ADHD = ADHD symptoms, Ext = Externalising. Only statistically significant paths are shown. Latent growth curves (inlcuding random intercepts that are allowed to covary and fixed slopes) and residual covariance parameters are omited for clarity

Model fit indices for the teacher-reported model including harsh parenting suggested good fit with RMSEA = 0.055, CFI = 0.984 and TLI = 0.911. In line with all other models, no significant mediation effects were identified. As in the parent-reported model, results suggested a significant cross-lagged effect from harsh parenting at age 9 to lower academic achievement at age 11. In addition, internalising problems at age 7 were associated with increased externalising problems at age 9. For a summary of significant autoregressive and cross-lagged effects, see Fig. 6. For indirect effects, see in Table S4(d) in the online supplementary and full results are available here: https://osf.io/34mge/.

### Sensitivity Analyses

The sensitivity analysis using a combined score of parent- and teacher- reported data on psychosocial difficulties yielded essentially the same results as the separate models with no significant mediation effects being identified neither when including parental involvement, nor when including harsh parenting. Full results of this analysis are available on OSF: https://osf.io/d7wfb/.

We also conducted additional analyses using a standard cross-lagged panel model in order to provide insights into why our results may have differed from previous studies. Cross-lagged panel models have been the method of choice in much of the previous longitudinal developmental psychopathology literature (e.g., Obsuth et al., 2020). However, their autoregressive and cross-lagged effects present a mixture of within- and between-person effects, thus, potentially leading to misleading results (Hamaker et al., 2015). The results of the parent-reported CLPM model including parental involvement as well as the model including harsh parenting suggested that a cascade from ADHD symptoms at age 7 to externalising problems at age 11 was significantly mediated by peer problems at age 9. The teacher-reported model including parental involvement further found support for mediating effects of peer problems and academic achievement in cascades from ADHD to internalising problems while the teacher reported model including harsh parenting suggested that academic achievement further mediates a cascade from ADHD to externalising problems. These differences in results highlight the need for appropriately disaggregating within- from between-person effects. The full model results for parent-and teacher-reported models can be found here: https://osf.io/d7wfb/

## Discussion

The aim of the current study was to comprehensively evaluate whether developmental cascades from externalising to internalising problems and from ADHD symptoms to internalising and externalising problems were mediated by parental involvement, harsh parenting, peer problems and academic underachievement when appropriately disaggregating within- from between-person effects. Results indicated that none of these factors acted as significant mediators when appropriately disaggregating within- and between-person effects. We also found only limited support for developmental cascades between the studied domains of psychosocial functioning. The baseline parent-reported model suggested reciprocal relations between internalising and ADHD symptoms across all studied time points as well as a cascade from ADHD at age 7 to externalising problems which in turn cascaded on ADHD symptoms at age 11. These cascades however were not observed in the baseline teacher-reported model which only suggested that internalising and externalising problems shared reciprocal relations between ages 7 and 9. The differences in observed developmental cascades across teachers and parents thus suggest that the context in which behaviours occur in is important for their effects on the development of further difficulties. When introducing potential mediators to the model, the observed teacher-reported cascades as well as the parent-reported cascade from internalising problems at age 7 to ADHD symptoms at age 9 were, however, no longer significant. This indicates that some of the variance explained in the baseline models through significant developmental cascades is shared with investigated mediating factors, albeit to an extent that was not detectable as significant mediation effects.

While many previous studies have found evidence for the dual failure model (e.g., van Lier et al., 2012), this is not the first study to report null-findings. For example, Poirier et al. (2019) found no evidence for a mediating effect of academic skills on developmental cascades from conduct problems to depressive symptoms. Similarly, in their study of 912 1^st^, 2^nd^ and 3^rd^ graders, Evans and Fite (2019) found only limited and mixed support for the dual failure model in linking aggressive behaviour to depressive symptoms over time. These authors concluded that the pathways via peer rejection and academic performance may be observed in younger children but not in children at later elementary school age. The only other study to date investigating mediating factors in externalising-internalising cascades also using an ALT-SR which appropriately accounts for between-person effects (Curran et al., 2014) also found no evidence for peer relationships or teacher relationships to mediate developmental cascades from aggression to internalising problems (Murray et al., 2021). Using data from the same cohort as the current study, but with a focus on adolescence (ages 11, 13, and 15), Murray et al. also did not identify any significant developmental cascades from aggression to internalising problems. This could indicate that previously identified developmental cascades and mediating relations are potentially not due to within-person processes but could have been the result of between-person confounding. In fact, the comparison of results with a standard CLPM in the current study also showed that when conflating within- and between-person effects, some of the hypothesised mediators were indeed identified as significant. In particular, the CLPM suggested that peer problems and academic achievement mediated developmental cascades from ADHD to externalising problems and ADHD symptoms to internalising problems. Nevertheless, there has also been some research showing evidence for externalising-internalising and ADHD-internalising cascades when appropriately accounting for between-person effects (Flouri et al., 2019; Murray et al., 2016, 2020a; Oh et al., 2020). However, studies using these methodologies account for only a minority of developmental cascade studies and more research using appropriate statistical tools to disaggregate within- and between-person effects are needed to evaluate developmental cascades and their potential mediators.

Looking specifically at the effect of parenting factors on the development of mental health problems, we observed two slightly counterintuitive effects in the parent-reported model. In particular, parental involvement was associated with increases in externalising problems whereas harsh parenting was associated with decreases in internalising problems. Prior research has suggested that coercive family processes are associated with an escalation of both internalising and externalising difficulties (Patterson, 2002; Speyer et al., 2022), whereas our findings using parent-reported data on internalising and externalising problems suggested the opposite effect. Considering that we did not observe the same associations in the teacher-reported model, one reason for these findings may be that parents using harsh parenting techniques may be less perceptive of their child’s internalising difficulties and thus underreport their emotional problems, whereas parents who are very involved in their children’s lives may overreport instances of externalising behaviours as they are very attuned to their child’s behaviour. A number of prior studies have highlighted that teacher and parent-reports are limited in their agreement on children’s psychosocial development (e.g. Cheng et al., 2018; Murray et al., 2018) with cross-informant correlation having been shown to average 0.25 for internalising problems, 0.30 for externalising problems (De Los Reyes, 2013) and range from 0.09 to 0.52 for ADHD symptoms (Narad et al., 2015). Cross-informant correlations in the present study were in line with these findings (0.11 to 0.22 for internalising problems,0.19 to 0.23 for externalising problems, 0.36 to 0.42 for ADHD symptoms). These discrepant findings highlight the need for a multi-informant perspective in developmental research as the nature of such discrepancies is currently not well understood.

The current study offered some evidence for developmental cascades from ADHD symptoms to internalising and externalising problems; however, these cascades were again not robust across informants. One potential reason for the discrepancy found here could be that parents and teachers observe children in different contexts, with some difficulties likely being tied to specific environments. In particular, findings suggest that ADHD symptoms observed by parents in the home context are more likely to be associated with the development of parent-observed internalising problems. ADHD symptoms tend to be picked up in the school context as this is where they primarily lead to problems (Murray et al., 2019c), thus, ADHD symptoms that are noticeable even in the home context may be particularly severe and therefore more likely to lead to the development of internalising problems than ADHD symptoms that are only problematic in the school context. Externalising problems, on the other hand, were associated with teacher-observed internalising problem, thus, potentially suggesting that externalising problems occurring in the school context are a stronger risk factor for the development of internalising problems than if such behaviours are picked up in the home context. This could potentially be because aggressive behaviours at school may interfere more with peer problems than if occurring in the home context. This would be in line with our finding that the significant cross-lagged effect from externalising to internalising problems was attenuated once we introduced mediators to our model. One other reason for the observed discrepancy could relate to the fact the questionnaire items in the parent-reported SBQ were presented in a randomised order whereas they were presented in the same order to all teachers. This would have addressed potential item position effects in the parents but not teachers.

### Strengths and Limitations

The key strength of this study is that we used a method suitable of overcoming the main limitation of previous studies investigating the dual failure model, that is an ALT-SR, which allowed us to separate within- from between-person effects (Curran et al., 2014). In addition, while most previous studies focused on one focal mediator and two domains of psychosocial functioning, we jointly modelled internalising, externalising and ADHD symptom cascades and potential mediators, thus, capturing the most commonly occurring and co-occurring conditions in childhood within one comprehensive model that unites the dual failure model with aspects of the ontogenic process model and Patterson’s coercion model. We were also able to investigate the studied cascades using both parent- and teacher-reported data on children’s psychosocial development, hence, providing a more complete picture of their development than when only using a single-informant approach.

However, the current study also has to be interpreted in light of some limitations. First, we relied on a community sample. While this has some advantages such as minimising the risk for Berkson’s bias (i.e. the overestimation of symptom co-occurrence; Berkson, 1946), it is possible that the developmental relations studied here would unfold differently in a clinical sample, thus future studies are needed. Second, the developmental period studied here was limited to middle childhood. While this is an important period from the perspective that both peers and parents are influential, it is possible that some of the investigated mediators are more prominent in different developmental periods. In particular, parent–child interactions as implicated in coercive family processes have been suggested to be particular central to the preschool years (Smith et al., 2014) whereas peer problems might be more relevant in adolescence when peer relationships become increasingly important (Brown & Larson, 2009). Future studies examining developmental cascades and potential mediators across early childhood to late adolescence would consequently be highly valuable. Third, the same broadband scale (i.e. the SBQ) was used to measure all mental health problems. This makes our analyses prone to a higher degree of shared method variance than if different, more specific scales for each individual mental health construct had been used. In particular, similarities between items belonging to different SBQ subscales could have primed participants to also respond similarly. This can potentially lead to an increase in Type I errors as such response patterns would increase the correlation between two different SBQ subscales. However, the precise impact of this is difficult to predict because the inflation of some parameters (e.g., autoregressive effects) could also contribute to the under-estimation of others (e.g., cross-lagged effects involving the same variable) and thus also increase Type II error rates in parts of the model. Fourth, the included measures were relatively brief. The measure of academic achievement consisted of only two items that were based on a teacher comparing students’ performance on mathematics and language skills to other students. Thus, it does not provide an objective indication of a child’s academic achievement. It is further possible that the lack of identified longitudinal relations between peer problems and internalising difficulties is due to the fact that only specific aspects of peer problems that were not captured in our measures are related to the development of internalising difficulties. Also, at some of the included time points, the measures of peer problems, parental involvement and harsh parenting had slightly lower reliability than desired (Cronbach’s alphas < 0.70) which could have attenuated their relation with other measures. We also used time intervals of two years to investigate developmental cascades. Shorter time lags could have provided different results as these cascades might act over shorter periods. Future studies should address this limitation by employing shorter time intervals to the study of mediating factors in developmental cascades of mental health problems. Experience sampling methods could be a useful approach to gain insights into the short-term relations between internalising problems, externalising problems, ADHD symptoms and social and academic failure. This could provide valuable clarification on which difficulties act as antecedents to other difficulties and consequently inform interventions. Finally, while ALT-SRs address between-person confounds that tend to be stable over time they are vulnerable to time-varying confounding. Future evaluations that can account for these and other complementary work using, for example, causal inference frameworks such as mendelian randomisation or matching-based methods could provide further insights into which mental health and psychosocial issues have a causal impact on others during development.

## Conclusion

Overall, the current study offered some of the most comprehensive insights into the relations between different mental health problems and hypothesised mediators in the developmental cascades literature to date. Findings of the main analysis do not provide support for the dual failure model, neither for the original hypothesised mediating role of peer problems and academic underachievement, nor for parental involvement as an additional potential mediator. Instead, the results highlight the importance of using statistical techniques that appropriately disaggregate within- from between-person effects. The sensitivity analyses using the more common cross-lagged panel model that confounds such effects offered some support for the hypothesised pathways of the dual failure model, thus indicating that some of the prior evidence for the dual failure model may be confounded by between-person effects. Considering the limitations of the current study and that it is one of very few studies appropriately disentangling within- from between-person effects when investigating mediating factors in developmental cascade models, further research is needed.

## Supplementary Information

Below is the link to the electronic supplementary material.

Supplementary file1 (DOCX 44 KB)

## Data Availability

Data available upon request.

## Code Availability

Code available upon request.
