# Supplementary material for: Mediating Factors in Within-Person Developmental Cascades of Externalising, Internalising and ADHD Symptoms in Childhood
Source: Res Child Adolesc Psychopathol. 2022 Apr 30;50(8):1011–25. doi: 10.1007/s10802-022-00905-5 (PMC9395455; doi:10.1007/s10802-022-00905-5)
Supplement: Supplementary file 1 — Supplementary file1 (DOCX 44 KB) [file 10802_2022_905_MOESM1_ESM.docx]

**Supplementary Materials**

| **Table S1.** Sample demographic information at baseline (age 7) | | | |
| --- | --- | --- | --- |
| **Variable** | **Category** | **%** | **N** |
| **Child Sex** | Female | 48.1 | 596 |
|  | Male | 51.9 | 644 |
| **Language Spoken at Home:** | Swiss German | 50.2 | 624 |
| **Child** | Other | 49.8 | 616 |
| **Language Spoken at Home**: | Swiss German | 37.5 | 384 |
| **Male Primary Caregiver** | Other | 62.5 | 640 |
| **Language Spoken at Home:** | Swiss German | 37.5 | 458 |
| **Female Primary Caregiver** | Other | 62.5 | 762 |
| **Country of Birth:** | Switzerland | 89.4 | 1109 |
| **Child** | Other | 10.6 | 131 |
| **Country of Birth:** | Switzerland | 40.7 | 418 |
| **Male Primary Caregiver** | Serbia-Montenegro | 10.4 | 107 |
|  | Germany | 4.2 | 43 |
|  | Portugal | 5.4 | 55 |
|  | Middle/Far East | 2.8 | 29 |
|  | Turkey | 5.0 | 51 |
|  | Other | 31.5 | 323 |
| **Country of Birth:** | Switzerland | 42.3 | 517 |
| **Female Primary Caregiver** | Serbia-Montenegro | 9.3 | 114 |
|  | Germany | 5.7 | 69 |
|  | Portugal | 4.8 | 59 |
|  | Middle/Far East | 4.8 | 59 |
|  | Turkey | 3.8 | 47 |
|  | Other | 29.3 | 365 |
| **Academic Qualification:** | Mandatory school or less | 21.0 | 213 |
| **Male Primary Caregiver** | Apprenticeship | 27.2 | 276 |
|  | A-levels | 9.7 | 99 |
|  | Higher vocational education | 17.1 | 174 |
|  | University | 25.0 | 254 |
| **Academic Qualification:** | Mandatory school or less | 25.4 | 309 |
| **Female Primary Caregiver** | Apprenticeship | 35.2 | 428 |
|  | A-levels | 7.8 | 95 |
|  | Higher vocational education | 15.5 | 189 |
|  | University | 16.0 | 195 |
|  |  | **Mean** | **SD** |
| **Child Age at Data Collection** | Age 7 Wave | 7.03 | 0.40 |
|  | Age 9 Wave | 8.93 | 0.38 |
|  | Age 11 Wave | 11.03 | 0.39 |

| **Table S2.** Descriptive statistics | | | | | | | | | | | | | | | | | | |
| --- | --- | --- | --- | --- | --- | --- | --- | --- | --- | --- | --- | --- | --- | --- | --- | --- | --- | --- |
|  |  | ***Age 7*** | | | | |  | ***Age 9*** | | | | |  | ***Age 11*** | | | | |
|  | ***N*** | ***Mean*** | ***SD*** | ***Var*** | ***Min*** | ***Max*** | ***N*** | ***Mean*** | ***SD*** | ***Var*** | ***Min*** | ***Max*** | ***N*** | ***Mean*** | ***SD*** | ***Var*** | ***Min*** | ***Max*** |
| Internalising (teacher) | 1302 | 1.86 | 0.76 | 0.58 | 1.00 | 5.00 | 1281 | 1.84 | 0.74 | 0.55 | 1.00 | 5.00 | 1034 | 1.90 | 0.76 | 0.58 | 1.00 | 5.00 |
| Internalising (parent) | 1201 | 1.61 | 0.49 | 0.24 | 1.00 | 3.86 | 1166 | 1.78 | 0.53 | 0.28 | 1.00 | 3.71 | 1064 | 1.84 | 0.57 | 0.32 | 1.00 | 4.29 |
| ADHD symptoms (teacher) | 1299 | 2.24 | 0.99 | 0.98 | 1.00 | 5.00 | 1281 | 2.06 | 0.95 | 0.90 | 1.00 | 5.00 | 1053 | 2.07 | 0.99 | 0.98 | 1.00 | 5.00 |
| ADHD symptoms (parent) | 1186 | 2.20 | 0.67 | 0.45 | 1.00 | 4.75 | 1155 | 2.29 | 0.70 | 0.49 | 1.00 | 4.75 | 1062 | 2.26 | 0.72 | 0.52 | 1.00 | 4.75 |
| Externalising (teacher) | 1263 | 1.48 | 0.57 | 0.32 | 1.00 | 4.35 | 1240 | 1.48 | 0.56 | 0.31 | 1.00 | 4.00 | 1039 | 1.40 | 0.52 | 0.27 | 1.00 | 4.06 |
| Externalising (parent) | 1087 | 1.58 | 0.37 | 0.14 | 1.00 | 3.71 | 1102 | 1.63 | 0.38 | 0.14 | 1.00 | 3.18 | 1022 | 1.59 | 0.39 | 0.15 | 1.00 | 3.53 |
| Peer problems | 1321 | 1.68 | 0.62 | 0.38 | 1.00 | 4.00 | 1285 | 1.68 | 0.67 | 0.45 | 1.00 | 4.50 | 1057 | 1.72 | 0.67 | 0.45 | 1.00 | 4.50 |
| Academic achievement | 1338 | 3.23 | 1.01 | 1.02 | 1.00 | 5.00 | 1279 | 3.21 | 1.01 | 1.02 | 1.00 | 5.00 | 1061 | 3.28 | 1.19 | 1.42 | 1.00 | 5.00 |
| Parental involvement | 1229 | 3.19 | 0.42 | 0.18 | 1.50 | 4.00 | 1180 | 3.08 | 0.41 | 0.17 | 1.10 | 4.00 | 1072 | 3.02 | 0.43 | 0.19 | 1.20 | 4.00 |
| Harsh parenting | 1229 | 1.45 | 0.49 | 0.24 | 1.00 | 3.67 | 1180 | 1.35 | 0.46 | 0.22 | 1.00 | 3.67 | 1072 | 1.25 | 0.43 | 0.19 | 1.00 | 3.33 |

| **Table S3.** Correlation matrix of observed variables | | | | | | | | | | | | | | | | | | | | | | | | | | | |
| --- | --- | --- | --- | --- | --- | --- | --- | --- | --- | --- | --- | --- | --- | --- | --- | --- | --- | --- | --- | --- | --- | --- | --- | --- | --- | --- | --- |
|  | 1. | 2. | 3. | 4. | 5. | 6. | 7. | 8. | 9. | 10. | 11. | 12. | 13. | 14. | 15. | 16. | 17. | 18. | 19. | 20. | 21. | 22. | 23. | 24. | 25. | 26. | 27. |
| 1. Age 7 internalising (teacher) | **-** |  |  |  |  |  |  |  |  |  |  |  |  |  |  |  |  |  |  |  |  |  |  |  |  |  |  |
| 2. Age 9 internalising (teacher) | **.50** | **-** |  |  |  |  |  |  |  |  |  |  |  |  |  |  |  |  |  |  |  |  |  |  |  |  |  |
| 3. Age 11 internalising (teacher) | **.24** | **.26** | **-** |  |  |  |  |  |  |  |  |  |  |  |  |  |  |  |  |  |  |  |  |  |  |  |  |
| 4. Age 7 internalising (parent) | **.11** | **.03** | **.04** | **-** |  |  |  |  |  |  |  |  |  |  |  |  |  |  |  |  |  |  |  |  |  |  |  |
| 5. Age 9 internalising (parent) | **.07** | **.12** | **.12** | **.53** | **-** |  |  |  |  |  |  |  |  |  |  |  |  |  |  |  |  |  |  |  |  |  |  |
| 6. Age 11 internalising (parent) | **.06** | **.10** | **.22** | **.43** | **.55** | **-** |  |  |  |  |  |  |  |  |  |  |  |  |  |  |  |  |  |  |  |  |  |
| 7. Age 7 ADHD (teacher) | **.40** | **.28** | **.20** | .05 | .07 | **.12** | **-** |  |  |  |  |  |  |  |  |  |  |  |  |  |  |  |  |  |  |  |  |
| 8. Age 9 ADHD (teacher) | **.26** | **.45** | **.13** | -.03 | .02 | **.06** | **.65** | **-** |  |  |  |  |  |  |  |  |  |  |  |  |  |  |  |  |  |  |  |
| 9. Age 11 ADHD (teacher) | **.21** | **.22** | **.37** | -.00 | **.09** | **.12** | **.53** | **.53** | **-** |  |  |  |  |  |  |  |  |  |  |  |  |  |  |  |  |  |  |
| 10. Age 7 ADHD (parent) | **.11** | **.09** | **.15** | **.38** | **.29** | **.28** | **.36** | **.27** | **.31** | **-** |  |  |  |  |  |  |  |  |  |  |  |  |  |  |  |  |  |
| 11. Age 9 ADHD (parent) | **.08** | **.12** | **.18** | **.26** | **.39** | **.33** | **.33** | **.34** | **.36** | **.65** | **-** |  |  |  |  |  |  |  |  |  |  |  |  |  |  |  |  |
| 12. Age 11 ADHD (parent) | **.09** | **.17** | **.18** | **.18** | **.29** | **.45** | **.34** | **.36** | **.42** | **.61** | **.69** | **-** |  |  |  |  |  |  |  |  |  |  |  |  |  |  |  |
| 13. Age 7 externalising (teacher) | **.37** | **.28** | **.12** | .05 | **.02** | **.03** | **.63** | **.49** | **.41** | **.26** | **.19** | **.23** | **-** |  |  |  |  |  |  |  |  |  |  |  |  |  |  |
| 14. Age 9 externalising (teacher) | **.24** | **.41** | **.08** | -.03 | .01 | **.06** | **.45** | **.69** | **.37** | **.16** | **.21** | **.24** | **.59** | **-** |  |  |  |  |  |  |  |  |  |  |  |  |  |
| 15. Age 11 externalising (teacher) | **.06** | **.10** | **.33** | -.02 | .02 | **.02** | **.31** | **.30** | **.59** | **.14** | **.14** | **.18** | **.39** | **.33** | **-** |  |  |  |  |  |  |  |  |  |  |  |  |
| 16. Age 7 externalising (parent) | -.01 | -.02 | .02 | **.37** | **.24** | **.26** | **.15** | **.16** | **.13** | **.47** | **.40** | **.35** | **.19** | **.14** | **.15** | **-** |  |  |  |  |  |  |  |  |  |  |  |
| 17. Age 9 externalising (parent) | -.01 | .05 | **.11** | **.25** | **.35** | **.30** | **.15** | **.18** | **.15** | **.37** | **.53** | **.39** | **.12** | **.17** | **.16** | **.67** | **-** |  |  |  |  |  |  |  |  |  |  |
| 18. Age 11 externalising (parent) | .01 | **.08** | **.11** | **.22** | **.27** | **.38** | **.16** | **.19** | **.20** | **.35** | **.44** | **.47** | **.15** | **.21** | **.23** | **.58** | **.75** | **-** |  |  |  |  |  |  |  |  |  |
| 19. Age 7 peer problems | **.37** | **.25** | **.17** | .08 | **.05** | **.09** | **.41** | **.29** | **.25** | **.19** | **.14** | **.20** | **.57** | **.36** | **.21** | **.07** | .05 | **.12** | **-** |  |  |  |  |  |  |  |  |
| 20. Age 9 peer problems | **.27** | **.46** | **.18** | .01 | **.11** | **.10** | **.37** | **.42** | **.30** | **.19** | **.16** | **.19** | **.38** | **.52** | **.19** | .02 | **.08** | **.12** | **.45** | **-** |  |  |  |  |  |  |  |
| 21. Age 11 peer problems | **.17** | **.18** | **.54** | **.07** | **.07** | **.15** | **.23** | **.20** | **.39** | **.25** | **.15** | **.21** | **.26** | **.22** | **.55** | **.16** | **.14** | **.21** | **.20** | **.23** | **-** |  |  |  |  |  |  |
| 22. Age 7 academic achievement | **-.21** | **-.13** | **-.11** | -.07 | -.05 | **-.10** | **-.37** | **-.24** | **-.26** | **-.15** | **-.17** | **-.18** | **-.17** | **-.11** | -.04 | **.05** | **.09** | .03 | **-.26** | **-.19** | **-.09** | **-** |  |  |  |  |  |
| 23. Age 9 academic achievement | **-.16** | **-.23** | **-.15** | -.02 | -.09 | -.09 | **-.26** | **-.28** | **-.26** | -.09 | **-.14** | **-.14** | **-.08** | **-.10** | .02 | **.10** | **.06** | **.06** | **-.07** | **-.19** | **-.11** | **.56** | **-** |  |  |  |  |
| 24. Age 11 academic achievement | **-.17** | **-.15** | **-.28** | .02 | -.03 | **-.07** | **-.32** | **-.28** | **-.42** | **-.14** | **-.17** | **-.24** | **-.17** | **-.14** | **-.11** | **.10** | **.09** | **.05** | **-.16** | **-.18** | **-.17** | **.58** | **.59** | **-** |  |  |  |
| 25. Age 7 parental involvement | **-.07** | -.06 | .02 | **-.13** | **-.13** | **-.09** | -.07 | -.**09** | -.06 | **-.10** | -.08 | -.07 | -.02 | -.07 | -.03 | **-.19** | **-.13** | **-.14** | .03 | .01 | -.02 | **.05** | .03 | .02 | **-** |  |  |
| 26. Age 9 parental involvement | -.02 | .02 | -.02 | **-.05** | **-.17** | **-.08** | .04 | -.00 | -.06 | -.06 | **-.10** | **-.06** | .00 | -.00 | -.05 | **-.24** | **-.22** | **-.16** | .04 | .02 | -.02 | -.08 | **-.06** | **-.09** | **.61** | **-** |  |
| 27. Age 11 parental involvement | -.02 | .00 | **-.03** | **-.09** | **-.18** | **-.16** | .01 | .01 | -.03 | **-.13** | **-.18** | **-.14** | .01 | -.03 | -.00 | **-.25** | **-.25** | **-.28** | -.02 | .04 | -.01 | -.02 | -.02 | -.08 | **.54** | **.65** | **-** |

*Note.* Correlations in bold are significant at *p* < .05

| **Table S4.** Indirect effects | | | | | |
| --- | --- | --- | --- | --- | --- |
| **(a) Parent-reported ALT-SR - involvement** | ***β*** | ***SE*** | ***p*** | ***CI_lower_*** | ***CI_upper_*** |
| Age 7 externalising to age 11 internalising |  |  |  |  |  |
| via age 9 academic achievement | 0.007 | 0.010 | .489 | -0.008 | 0.038 |
| via age 9 peer problems | 0.006 | 0.009 | .464 | -0.009 | 0.030 |
| via age 9 parental involvement | -0.000 | 0.005 | .948 | -0.021 | 0.014 |
| sum of indirect effect | 0.013 | 0.014 | .365 | -0.018 | 0.059 |
| Age 7 ADHD to age 11 internalising |  |  |  |  |  |
| via age 9 academic achievement | 0.007 | 0.011 | .501 | -0.009 | 0.056 |
| via age 9 peer problems | -0.002 | 0.004 | .620 | -0.014 | 0.007 |
| via age 9 parental involvement | 0.000 | 0.001 | .958 | -0.012 | 0.014 |
| sum of indirect effect | 0.005 | 0.011 | .642 | -0.018 | 0.058 |
| Age 7 ADHD to age 11 externalising |  |  |  |  |  |
| via age 9 academic achievement | 0.019 | 0.018 | .278 | -0.004 | 0.091 |
| via age 9 peer problems | 0.002 | 0.005 | .641 | -0.007 | 0.016 |
| via age 9 parental involvement | 0.000 | 0.001 | .923 | -0.012 | 0.019 |
| sum of indirect effect | 0.021 | 0.018 | .232 | -0.007 | 0.099 |
| **(b) Teacher-reported ALT-SR - involvement** | ***β*** | ***SE*** | ***p*** | ***CI_lower_*** | ***CI_upper_*** |
| Age 7 externalising to age 11 internalising |  |  |  |  |  |
| via age 9 academic achievement | -0.000 | 0.004 | .771 | -0.014 | 0.011 |
| via age 9 peer problems | 0.001 | 0.003 | .825 | -0.009 | 0.013 |
| via age 9 parental involvement | 0.005 | 0.008 | .474 | -0.007 | 0.025 |
| sum of indirect effect | 0.005 | 0.009 | .599 | -0.014 | 0.032 |
| Age 7 ADHD to age 11 internalising |  |  |  |  |  |
| via age 9 academic achievement | 0.000 | 0.012 | .985 | -0.013 | 0.013 |
| via age 9 peer problems | 0.005 | 0.007 | .442 | -0.008 | 0.022 |
| via age 9 parental involvement | -0.012 | 0.011 | .274 | -0.038 | 0.009 |
| sum of indirect effect | -0.007 | 0.014 | .620 | -0.040 | 0.024 |
| Age 7 ADHD to age 11 externalising |  |  |  |  |  |
| via age 9 academic achievement | 0.000 | 0.008 | .985 | -0.024 | 0.032 |
| via age 9 peer problems | 0.006 | 0.008 | .455 | -0.022 | 0.014 |
| via age 9 parental involvement | 0.017 | 0.020 | .403 | -0.009 | 0.073 |
| sum of indirect effect | 0.011 | 0.023 | .629 | -0.025 | 0.075 |
| **(c) Parent-reported ALT-SR - harsh** | ***β*** | ***SE*** | ***p*** | ***CI_lower_*** | ***CI_upper_*** |
| Age 7 externalising to age 11 internalising |  |  |  |  |  |
| via age 9 academic achievement | 0.003 | 0.006 | .620 | -0.015 | 0.026 |
| via age 9 peer problems | 0.001 | 0.007 | .841 | -0.019 | 0.022 |
| via age 9 harsh parenting | -0.008 | 0.008 | .360 | -0.045 | 0.004 |
| sum of indirect effect | -0.053 | 0.014 | .819 | -0.059 | 0.035 |
| Age 7 ADHD to age 11 internalising |  |  |  |  |  |
| via age 9 academic achievement | 0.006 | 0.012 | .620 | -0.023 | 0.039 |
| via age 9 peer problems | 0.000 | 0.002 | .862 | -0.011 | 0.006 |
| via age 9 harsh parenting | -0.010 | 0.009 | .268 | -0.038 | 0.005 |
| sum of indirect effect | -0.004 | 0.016 | .784 | -0.053 | 0.036 |
| Age 7 ADHD to age 11 externalising |  |  |  |  |  |
| via age 9 academic achievement | 0.024 | 0.019 | .205 | -0.006 | 0.080 |
| via age 9 peer problems | 0.001 | 0.003 | .727 | -0.006 | 0.017 |
| via age 9 harsh parenting | -0.012 | 0.010 | .253 | -0.043 | 0.009 |
| sum of indirect effect | 0.013 | 0.021 | .543 | -0.031 | 0.077 |
| **(b) Teacher-reported ALT-SR - harsh** | ***β*** | ***SE*** | ***p*** | ***CI_lower_*** | ***CI_upper_*** |
| Age 7 externalising to age 11 internalising |  |  |  |  |  |
| via age 9 academic achievement | 0.001 | 0.004 | .756 | -0.011 | 0.017 |
| via age 9 peer problems | 0.000 | 0.003 | .892 | -0.010 | 0.011 |
| via age 9 harsh parenting | 0.001 | 0.003 | .836 | -0.008 | 0.011 |
| sum of indirect effect | 0.002 | 0.006 | .713 | -0.016 | 0.025 |
| Age 7 ADHD to age 11 internalising |  |  |  |  |  |
| via age 9 academic achievement | -0.003 | 0.005 | .553 | -0.018 | 0.011 |
| via age 9 peer problems | 0.006 | 0.007 | .443 | -0.007 | 0.026 |
| via age 9 harsh parenting | -0.001 | 0.003 | .834 | -0.009 | 0.010 |
| sum of indirect effect | 0.002 | 0.010 | .826 | -0.022 | 0.024 |
| Age 7 ADHD to age 11 externalising |  |  |  |  |  |
| via age 9 academic achievement | 0.009 | 0.014 | .538 | -0.024 | 0.048 |
| via age 9 peer problems | -0.006 | 0.008 | .455 | -0.023 | 0.016 |
| via age 9 harsh parenting | -0.001 | 0.004 | .822 | -0.011 | 0.018 |
| sum of indirect effect | 0.002 | 0.017 | .915 | -0.028 | 0.051 |
| *Note.* Confidence Intervals (CIs) are based on 1000 bootstrapped samples using standard maximum likelihood estimation | | | | | |
